# Conjoined NO-sensitive guanylyl cyclases

**DOI:** 10.1186/1471-2210-11-S1-P6

**Published:** 2011-08-01

**Authors:** Sönke Behrends, Mareike Busker, Nadine Haase, Tobias Haase, Jan Krähling, Monika Linnenbaum

**Affiliations:** 1 Institut für Pharmakologie, Toxikologie und Klinische Pharmazie, TU Braunschweig, Germany

## Background

NO-sensitive guanylyl cyclases (NO-GC’s) that catalyze the reaction of GTP to the second messenger molecule cGMP are heterodimeric enzymes consisting of an α and a β_1_ subunit. The two prevalent isoforms in humans (α_1_/β_1_, α_2_/β_1_) are time-tested targets for drugs that release nitric oxide (NO) and new compounds that either sensitize the enzyme for activation by NO or activate the enzyme independently of NO. Homologous NO-GC subunits consist of an amino-terminal HNOX domain followed by a PAS- and a coiled coil domain and the carboxy-terminal catalytic domain.

## Results

Measurement of FRET between fluorescent proteins tagged to the amino- and carboxy-terminus of the subunits indicated close proximity between the amino-terminal HNOX domains and the carboxy-terminal catalytic domains of the enzyme. On the basis of these results we constructed conjoined NO-GC’s by fusion of the α amino-terminus to the β_1_ carboxy-terminus leading to a monomeric enzyme complex. Surprisingly but in accordance with the FRET results these conjoined NO-GC’s (β_1_α_1_, β_1_α_2_) showed specific enzyme activity and stimulation by NO and various modulators of GC activity.

## Conclusion

These obligate enzyme variants faithfully reproduced the pharmacological properties of the heterodimeric enzymes including an isoform specific differential activation of the NO-independent drug cinaciguat. Novel applications of conjoined NO-GC’s including adenoviral gene transfer will be discussed.

